# Experiential Learning in Patient Safety: A Multi-Center Study Examining Emergency Medicine Residents' Situational Awareness of Hazards via Simulation

**DOI:** 10.7759/cureus.30648

**Published:** 2022-10-24

**Authors:** Kamna S Balhara, Nathan Olson, Jessica L Wilson, Rosemarie G Ramos, Harlan J Goode, Andrew E Muck, Adriana S Olson

**Affiliations:** 1 Emergency Medicine, Johns Hopkins University School of Medicine, Baltimore, USA; 2 Emergency Medicine, University of Chicago, Chicago, USA; 3 Emergency Medicine, University of Texas Health Science Center at Houston, Houston, USA; 4 Family and Community Medicine, University of Texas Health Science Center at San Antonio, San Antonio, USA; 5 Emergency Medicine, Saint David's Hospital, Austin, USA; 6 Emergency Medicine, University of Texas Health Science Center at San Antonio, San Antonio, USA

**Keywords:** emergency department, situational awareness, simulation, emergency medicine, patient safety

## Abstract

Introduction

The 2016 Clinical Learning Environment Review established that experiential patient safety curricula for residents are uncommon. Moreover, these curricula do not incorporate non-technical skills linked to safety, such as situational awareness (SA). We developed an in-situ patient safety simulation exercise incorporating core SA concepts and subsequently assessed exercise feasibility and acceptability, and measured residents' safety SA.

Methods

A simulation scenario and mock chart were designed, incorporating 16 patient safety hazards. Residents at two institutions reviewed the chart and had 10 minutes in an emergency department room with the simulated scenario to document identified hazards, followed by a facilitated debriefing. Pre- and post-exercise surveys were completed. We used regression analyses to compare exercise performance and survey responses by training year, and measures of proportional difference and association for survey responses.

Results

This study included 76 of 104 eligible residents (73.1%). Around 56.5% initially reported being comfortable identifying hazards. During the exercise, hazards requiring higher SA were identified less frequently. Senior residents identified more hazards (OR 2.26; 95%CI 1.56-3.28) (mean 8.28, SD1.45); 93.4% expressed satisfaction with the session, and residents reporting comfort increased significantly (89.5%, p<0.001).

Conclusion

In-situ simulation incorporating SA concepts feasibly provides experiential safety education and enhances resident comfort with safety issues. Visible hazards were often identified; those requiring information synthesis were usually missed, suggesting a need for developing more robust resident SA. While interns demonstrated the poorest performance, senior residents only identified 50% of errors, indicating that patient safety education enhancing SA should begin early and continue longitudinally.

## Introduction

National healthcare agencies and educational organizations have identified patient safety as a key intervention target across a broad range of clinical environments [1,2]. The emergency department (ED) is particularly susceptible to safety hazards, given large volumes of high-acuity patients, distractions, and interdisciplinary teams prone to circadian fatigue [3]. Hazards may lead to "adverse events" (4.1/100 patient visits), which constitute injuries or harms suffered by patients due to medical care, as well as "near misses" (5.4/100 patient visits), wherein an event could have had "adverse consequences but did not and was indistinguishable from fully fledged adverse events in all but outcome [4]." Adverse events may be preventable and are associated with recidivism and mortality [5,6].

Despite this, patient safety curricula targeting safety hazards remain deficient in graduate medical education (GME) and little is known about residents' awareness of safety hazards in clinical settings [7]. In 2016, the Clinical Learning Environment Review (CLER) reported that residents lack awareness of key safety concepts, and "while many Clinical Learning Environments (CLEs) provided didactic training in patient safety, it was uncommon for CLEs to provide . . . opportunities for experiential learning [2]." Studies involving nurses, pharmacists, medical students, and interns have demonstrated the feasibility of simulation (SIM) models to both understand existing trainee knowledge and enhance experiential patient safety learning [8-13]. However, implementation and evaluation of SIM-based patient safety exercises are lacking for residency trainees; a recent systematic review found that of 27 residency-based programs for teaching patient safety, none used SIM [14]. 

Additionally, despite growing recognition that non-technical skills (NTS) are of increasing importance in safe patient care [1], patient safety curricula explicitly incorporating non-technical skills (NTS) are limited. NTS are cognitive and social competencies that complement technical skills and permit safe and efficient communication, decision-making, and task performance [15]. Such skills have long been implicated in enhancing safety in high-risk industries such as aviation and the military. Specifically, situational awareness (SA), defined as "perception of elements in the environment within a volume of time and space, comprehension of their meaning, and projection of their status in the near future," is an integral NTS in improving patient safety in complex environments [1,15-18]. It has been suggested as a key skill in emergency medicine (EM) and may enhance physicians' ability to recognize and predict sources of harm. 

In anesthesia, surgery, critical care, and EM, SIM has been suggested as an ideal environment to address clinicians' SA [19-21]. However, SA is rarely incorporated into patient safety education in EM [1,15,16]. Moreover, SIM-based safety education is rarely conducted in the context of the clinical environment, often occurring instead in SIM centers, which do not always emulate the complexity of real clinical settings, such as ambient physical stressors, distractions, and clinical devices that can negatively impact SA [19,21-23].

Given the paucity of experiential curricula for GME patient safety education, the importance of SA in identifying hazards, and the potential utility of SIM in safety education and in heightening SA, we incorporated concepts of SA into a SIM-based patient safety exercise designed to assess and potentially enhance residents' SA of safety hazards in the clinical environment of the ED. 

Our objectives were to assess resident SA of patient safety in clinical settings and to evaluate the feasibility and acceptability of employing an in-situ SIM in patient safety education.

This work was presented as a poster presentation at the Society for Academic Emergency Medicine's Annual Meeting in May 2018 in Indianapolis, Indiana.

## Materials and methods

Study setting

This study was conducted with EM residents at two Accreditation Council for Graduate Medical Education (ACGME)-accredited three-year programs at tertiary care centers. Program 1 has 10 residents per post-graduate year (PGY); program 2 has 16 residents per PGY. Neither had an existing formal patient safety curriculum. Both institutions' ethical review boards approved this study as exempt as participation was part of each site's routine residency educational curriculum.

Study design

We conducted a prospective, observational study utilizing in-situ SIM with pre- and post-exercise surveys. Our primary objectives were to (1) obtain a baseline surrogate measure of residents' SA by tabulating how many hazards residents were able to identify in a simulated environment that mimicked some elements of their actual patient care environments, and (2) assess intervention feasibility and acceptability. Intervention impact on residents' comfort with hazard identification represented a secondary outcome.

Common and serious hazards to patient safety in local ED environments were identified by a panel of clinical leaders, including nursing staff and physicians, and supplemented by a literature review. Subsequently, 16 safety hazards of varying complexity deemed important to the local context and aligned with national patient safety priorities were identified [3,24]. Hazards were classified into levels of SA based on Endsley's model [18]. (Tables 1-3). Study investigators determined what constituted “correct” hazard identification and rectification (i.e., proposed solution).

**Table 1:** Endsley's levels of situational awareness SA: situational awareness [18]

Level of situational awareness (SA)	Description
Level 1: Perception of elements in the environment	Perceiving status, attributes, and dynamics of relevant elements in the environment
Level 2: Comprehension of current situation	Synthesizing disjointed level 1 elements from multiple sources to understand the significance of those elements in light of pertinent operator goals, to form patterns that contribute to a holistic picture of the environment
Level 3: Projection of future status	Projecting future action of environmental elements through knowledge of the status and dynamics of the elements and comprehension of the situation (i.e. Level 1 and 2 SA)

**Table 2:** Human and systems factors impacting individual situational awareness SA: situational awareness [18]

Human properties impacting situational awareness (SA)	Task and system factors impacting SA
Preattentive processing: initial parallel processing of environmental features through preattentive sensory stores, leading to identification of cues upon which to focus attention	System design: SA is influenced by the degree to which the system gains need information from the environment
Attention: a major limit on SA, as it limits operators' ability to perceive and process, multiple cues in parallel	Interface design: the manner in which information is presented
Perception: will be influenced by any pre-existing expectations about the information	Stress: includes physical, social, and psychological stressors
Working memory: permits active processing of information based upon existing knowledge, may be heavily taxed if limited long-term memory stores	Workload: significant stressor impacting SA
Long-term memory: structures that may circumvent limits of working memory, including mental models	Complexity: may negatively impact workload and SA
Confidence level: confidence pertaining to accuracy of information received as well as subsequent SA	Automation: automated processes may lead to diminished perception and SA
Automaticity: rapid, subconscious, and effortless processing that may overcome limited attention	
Goals: SA is linked with context and decisions for which SA is being sought	

**Table 3:** Hazards simulated, associated level of situational awareness, and proportion of residents identifying hazard correctly

Hazard type	Level of situational awareness	Number of residents correctly identifying hazards (%)
Hazards heightening fall risk in any patient		
High bed	1	67 (88.2)
Lowered bed rail	1	59 (77.6)
Patient not wearing non-skid/non-slip hospital-issue socks	1	35 (46.1)
Environmental hazards		
Overflowing trash basket	1	56 (73.4)
Exposed sharps in room	1	74 (97.4)
Call button out of patient’s reach	1/2	28 (36.8)
Foley catheter not placed to gravity	1	44 (57.9)
Patient misidentification hazard		
Patient lacking identification band	1	13 (17.1)
Medication-related hazards		
Unlabeled medication infusing via IV line	1	41 (54.0)
Medications in syringes left in room and not disposed of appropriately	1	44 (57.9)
Hazards specific to patient presentation and history		
Patient not placed on contact isolation for suspected C. difficile	2	28 (36.8)
Patient with suspected C. difficile and soap dispenser is empty	2	19 (25.0)
No bag valve mask in patient room	2/3	9 (11.8)
Food tray in room	2	33 (43.4)
Discrepancy between patient's allergy band and recorded allergies	2/3	18 (23.7)
Patient is a fall risk and not wearing fall bracelet	2	6 (7.9)

Hazards were then incorporated into a SIM scenario which included a mock patient chart and a simulated patient room set-up of the mock patient. The mock chart was integrated into the patient health record system used at each individual site (Supplemental Appendix 1). The SIM was conducted in an ED patient room not in use at that time. A SimMan® 3G mannequin (Laerdal Medical, Stavanger, Norway) and existing ED equipment, including monitors, infusion pumps, and computers, were utilized to simulate hazards; both sites had identical setups and pilot setups were photographed to ensure consistency. 

Surveys were designed to capture the acceptability and feasibility of the SIM as an educational intervention and its impact on resident comfort; the latter has been identified as a potential area of deficiency [25]. Face validity was confirmed via group consensus (Supplemental Appendix 2 and 3).

SIM sessions were incorporated into existing didactic time, lasted less than an hour, and could be completed with a single faculty facilitator. A five-minute-long pre-briefing using a standardized script was conducted by one facilitator (clinician-educator) at each site (Supplemental Appendix 4).

After a pre-intervention survey, residents independently reviewed the mock chart at a clinical workstation, while exposed to ambient stressors and sources of distraction, including noises inherent to the clinical environment. They then entered the room in groups of four to five, to minimize the need for scenario repetition, and were given 10 minutes to independently document identified hazards and proposed solutions during the exercise. They were instructed not to interact with each other or manipulate any objects. No new information was provided and no changes in patient status occurred once residents had entered the room. A researcher monitored the exercise to ensure protocol adherence. During a subsequent facilitator-led debriefing session using a semi-structured script, participants discussed identified hazards and reflected on potential solutions, then completed a post-exercise survey.

Analysis

Survey responses and scored lists of documented hazards were tabulated into Excel (Microsoft Office, 2017) by two independent reviewers, with discrepancies adjudicated by group consensus. Analyses were conducted using STATA 12.0 (Stata Corp, College Station, TX); all levels of significance were set at p=0.05.

Descriptive statistics were used for the frequency of hazards identified. Linear regression was utilized to assess whether training site, year-in-training, and pre-intervention comfort predicted exercise performance. Descriptive statistics were used to summarize survey responses. Correlations between training year and reported experience and comfort with hazards and the number of correct solutions proposed were assessed with logistic regression. Responses between sites were compared with chi-square tests. Two-sample tests of proportions were used to compare self-reported comfort pre- and post-exercise.

## Results

Participant characteristics

Around 76 of 104 (73.1%) eligible residents participated, including 21 PGY0s (starting training), 19 PGY1s (completed one year of training), 14 PGY2s (completed two years of training), and 22 PGY3s (completed three years of training). 

Prior experience with addressing hazards

In this study, 45 (59.2%) and 56 (73.3%) residents reported identifying at least one patient safety hazard in the ED in the last two months and last year, respectively. Increased years of training predicted self-reported hazard identification (OR 2.35; 95% CI, 1.47-3.76; OR 4.91; 95% CI, 2.19-11.04), but did not predict hazard rectification (OR 1.56; 95% CI, 0.95-2.57; OR 1.76; 95% CI, 0.99-3.13).

Pre-exercise comfort with addressing hazards

Around 43 (56.6%) and 33 (43.4%) respondents reported pre-exercise comfort with identifying and rectifying hazards, respectively. Self-reported degree of comfort with hazard identification and rectification increased modestly by training year (OR 1.78; 95% CI, 1.17-2.72; OR 1.55; 95% CI, 1.03-2.32). Comfort was not associated with the training site (p=0.58, p=0.14).

Hazards identified during exercise

Hazards necessitating a lower level of SA were most commonly identified (Table 3). PGY0s identified a mean of 5.86 hazards (SD 1.82), PGY1s 7.73 (SD 2.14), PGY2s 8.86 (SD 1.96), and PGY3s 8.28 (SD 1.45). Residents in higher years of training were significantly more likely to identify more hazards (OR 2.26; 95% CI, 1.56-3.28). Higher levels of reported pre-exercise comfort predicted the number of hazards identified (OR 3.19, 95% CI, 1.20-8.07). The training site did not impact hazard identification (OR 1.11; 95% CI, 0.45-2.64). There was no correlation between the year of training and the number of appropriate solutions proposed (OR 0.8; 95% CI, 0.53-1.2). 

During debriefing, most solutions discussed by residents included directly addressing the issue themselves (e.g. raising the bed rail while in the room) or instructing nursing or housekeeping staff to address the hazard. There were fewer proposed upstream solutions, such as improving team education and awareness of hazards, or systematic scheduled checks to ensure the availability of key supplies. 

Post-exercise comfort with addressing hazards

Residents reporting comfort with identifying and rectifying hazards increased significantly post-exercise to 68 (89.5%) and 64 (84.2%), respectively (p-value <0.001 at each site). Self-reported comfort post-exercise did not differ significantly by year in training (OR 1.98; 95% CI, 0.93-4.21; OR 1.55; 95% CI, 1.03-2.32). More than 80% reported satisfaction with the exercise (PGY0 18 (85.7%), PGY1 17 (89.5%), PGY2 13 (92.9%), PGY3 22 ((100%)). More than 80% agreed or strongly agreed that the exercise increased their knowledge of patient safety hazards (PGY0 17 (81.0%), PGY1 16 (84.2%), PGY2 13 (93.0%), PGY3 19 (86.4%)).

## Discussion

SA, which has been implicated in harm reduction in industries such as aviation, is especially relevant to safety in acute care medicine [15-17]. Our study of a SIM-based pilot intervention conducted in-situ in the ED identifies gaps in existing resident awareness of safety hazards at two sites and demonstrates that an in-situ SIM targeting safety hazards and grounded in concepts of SA is 1) feasible and acceptable to learners, and 2) may enhance resident comfort with addressing safety hazards.

In our study, incoming interns identified the fewest hazards. Patient safety curricula in medical schools are lacking; only 25% of American and Canadian medical schools report having such curricula. Of these, only 30% use SIM [26]. Moreover, junior trainees have limited clinical exposure and are vulnerable to cognitive overload and stress, which may contribute to a decreased ability to recognize critical cues (level 1 SA), limited working memory (level 2 SA), and projection of future courses of action (level 3 SA). While there are few substitutes for time spent in the clinical environment, SIM scenarios could serve as an important auxiliary tool for junior trainees to "increase" clinical experience, enhance cue recognition, and exercise working memory in clinical contexts. 

Senior residents demonstrated superior performance as compared to junior residents. Greater clinical exposure permits "recognition-primed" decision-making, allowing advanced trainees to match situations to learned patterns to make rapid accurate projections [17,18,27]. A prior study of EM residents in the clinical environment also demonstrated that increased years of training were associated with increased clinical SA [28]. However, in our study, senior residents on average only identified 50% of hazards, and identification rates plateaued amongst PGY2 and 3 residents. Senior residents may incorrectly apply patterns to situations or develop automatic processing, which makes them less responsive to new stimuli, for instance, changes in patient condition or monitors alarming [17,18]. Repeated comparisons of mental models with actual situations permit trainees to cross-check their mental models; longitudinally-scheduled SIM exercises may help ensure that senior residents are not applying inappropriate mental models to high-risk situations. 

Our findings also suggest that further development of level 2 and 3 SA in relation to patient safety is needed. Hazards that were wholly dependent on visual cue detection and simple identification of elements (level 1 SA), such as exposed sharps, were frequently identified. However, hazards mandating synthesis of perceived disparate elements (level 2 SA), i.e. visual cues (e.g. an allergy band on a patient's wrist) and chart information (e.g. absence of allergies listed in the chart), were less frequently identified across all PGYs. Hazards that demanded perception, synthesis, and subsequent anticipation of system vulnerabilities and patient deterioration (level 3 SA) were rarely identified (e.g. lack of bag-valve mask). While our findings are from a small sample size at two institutions, other studies corroborate the relative paucity of synthesis and anticipation skills of residents as they pertain to patient safety [29].

SIM represents an ideal instructional modality to maximize experiential learning and assess context-specific safety SA. Our study demonstrates that SIM-based interventions for patient safety education for residents can be reasonably performed in-situ with one facilitator using existing ED resources at a relatively low cost. Conducting the SIM within the clinical environment may also contribute to generating SA adapted to the complex spatial, auditory, and visual specifics of the trainees' clinical environment and allow interfacing with the electronic medical record (EMR) and existing clinical devices. Given the relative brevity of the exercise, it could be easily integrated into existing didactic time or clinical schedules, and may also lend itself to longitudinal curricula. Most participants expressed satisfaction with the intervention, indicating the acceptability of the format to learners. 

Participants also perceived increased comfort with identifying hazards in the clinical setting after participating in the SIM and debriefings. Debriefings provided a reflective period that could have contributed to trainee metacognition which may have contributed to enhanced comfort or SA. 

Successful implementation of this in-situ intervention was contingent on a number of factors, including the engagement of clinical stakeholders, such as medical directors and nursing leadership, who provided guidance on which hazards to incorporate and assisted with securing space and supplies. Integration of the activity into existing protected didactic times allowed us to obtain a robust number of participants for this intervention and also underscored the importance of the topic to residents. 

Limitations

Our study was subject to certain limitations. Residents' comfort level was self-reported and subject to bias, and recall bias may have impacted previously identified hazards reported. Our exercise likely overestimated residents' ability to recognize hazards, as the scenario did not replicate the full cognitive load and task burden of clinical shifts, and residents were primed to look for hazards prior to the exercise. Though residents were instructed not to interact, there is still a small possibility of peer influence or discussion through nonverbal cues.

The hazards chosen in this study were culled from national literature but adapted to local priorities. As such, specific study findings may not be generalizable. However, the intervention described here is easily adaptable to fit local needs.

## Conclusions

Our study suggests a need for enhancing EM residents' SA, especially at higher SA levels. Inferior performance at earlier stages of training indicates that safety education should be instituted early in residency; the plateau in senior residents' performance suggests that safety education should continue longitudinally. Our study also demonstrates that an in-situ SIM incorporating key concepts of SA is feasible, acceptable to learners, and may enhance learner comfort with addressing clinical hazards. The intervention described here is not resource-intensive and can be easily translated to other clinical sites to assess clinicians' SA or provide experiential patient safety education to trainees by adapting scenarios to site-specific needs and hazards. Our approach lays the groundwork for future studies that could examine educational and behavioral impacts by tracking hazard identification across longitudinal exercises and by monitoring clinical harm rates or residents' use of safety reporting mechanisms. 

To move beyond a baseline assessment of existing resident SA in static situations, future studies could also consider integrating tools such as the Situation Awareness Global Assessment Technique (SAGAT) to evaluate hazard awareness during the provision of simulated patient care or management of multiple simultaneous "patients," tasking residents to evaluate a patient and pausing the scenario to query residents about hazards. While the impact of such interventions on clinical outcomes is yet to be determined, the development of SA via in-situ SIM-based exercises could represent an important educational modality to assess and enhance the practice of safer clinical care.

## Appendices

Supplemental Appendix 1. Mock chart details 

Chief complaint at presentation: Diarrhea, fever 

Brief note by physician screening patient in triage area:

History of Present Illness: 

60 yo male with history of COPD, opiate abuse presents with 2 days of subjective fevers and diarrhea. He says he was recently in a hospital for pneumonia treated with antibiotics. He has had a lot of diarrhea, it’s watery and foul-smelling. He also feels lightheaded and has fallen multiple times at home. He generally can’t make it to the bathroom on time. 

Review of systems: 

As per above, patient otherwise does not indicate any further emergent/urgent complaints

Past medical history: COPD

Past surgical history: appendectomy

Medications: Albuterol inhaler as needed for wheezing, Spiriva daily, Methadone daily

Allergies: No known drug allergies 

Family history: non-contributory

Social history: Occasional alcohol use, current smoker (1 pack per day), former intravenous heroin use

Physical Exam:

Vital Signs: Blood pressure 145/70, Heart rate 80 beats per minute, Respiratory rate 18 breaths per minute, Oxygen saturation 98% on room air, Temperature 101F

General appearance: alert, appears fatigued

Head/Ears/Eyes/Nose/Oral/Throat: no evidence of trauma

Neck: Normal 

Cardiovascular: Regular rate and rhythm 

Respiratory: coarse breath sounds bilaterally with faint expiratory wheezes bilaterally

Abdomen: soft, nontender, nondistended

Extremities: no obvious lower extremity swelling

Neurological: Alert and oriented x 3, moves all extremities 

Skin: pale, cool 

Assessment/Plan: 

60 year old male with fever and large amounts of diarrhea, non-acute abdominal exam. Order laboratory tests, chest x-ray, urinalysis.

Supplemental Appendix 2: Pre-exercise Survey

Number on Card:

PGY Year: 

How many times in the last 2 weeks have you identified a safety hazard in the Emergency Department?

How many times in the last 2 weeks have you rectified a safety hazard in the Emergency Department?

How many times in the last 2 months have you identified a safety hazard in the Emergency Department?

How many times in the last 2 months have you rectified a safety hazard in the Emergency Department?

How many times in the last year have you identified a safety hazard in the Emergency Department?

How many times in the last year have you rectified a safety hazard in the Emergency Department?

On a scale from 1-5, with 1 being “very uncomfortable” and 5 being “very comfortable,” please rate your comfort level with identifying safety hazards in the Emergency Department. (Please circle for answer)

          1                                2                             3                             4                       5 Very                     Somewhat                  Neutral              Somewhat                Very 

Uncomfortable          Uncomfortable                                    Comfortable           Comfortable
 

On a scale from 1-5, with 1 being “very uncomfortable” and 5 being “very comfortable,” please rate your comfort level with rectifying safety hazards in the Emergency Department. (Please circle for answer)

      1                                2                             3                             4                       5 Very                     Somewhat                  Neutral              Somewhat                Very 

Uncomfortable          Uncomfortable                                    Comfortable           Comfortable

Supplemental Appendix 3: Post-exercise Survey

Number on Card:

On a scale from 1-5, with 1 being “very uncomfortable” and 5 being “very comfortable,” please rate your comfort level with identifying safety hazards in the Emergency Department. (Please circle for answer)

          1                             2                       3                            4                         5 Very                 Somewhat              Neutral           Somewhat                 Very 

Uncomfortable      Uncomfortable                              Comfortable          Comfortable

On a scale from 1-5, with 1 being “very uncomfortable” and 5 being “very comfortable,” please rate your comfort level with rectifying safety hazards in the Emergency Department. (Please circle for answer)

         1                             2                       3                            4                         5 Very                 Somewhat              Neutral           Somewhat                 Very 

Uncomfortable      Uncomfortable                              Comfortable          Comfortable

On a scale from 1-5, with 1 being “very unsatisfied” and 5 being “very satisfied,” please rate your satisfaction with the simulation session in the Emergency Department. (Please circle for answer)

          1                             2                       3                           4                          5 Very                   Somewhat            Neutral             Somewhat               Very      

   Unsatisfied           Unsatisfied                                      Satisfied                Satisfied 

On a scale from 1-5, with 1 being “Strongly disagree” and 5 being “Strongly agree,” please rate how strongly you agree that this exercise improved your knowledge of patient safety hazards in the Emergency Department. (Please circle for answer)

          1                              2                      3                            4                          5 Strongly                 Somewhat           Neutral              Somewhat             Strongly

   Disagree               Disagree                                             Agree                 Agree

Supplemental Appendix 4. Pre-briefing instructions

Verbal instructions were as follows: "You will be entering this ED room with the task of identifying patient safety hazards and will be given time afterward to formulate solutions. You will have a patient chart to read prior to entering the room. Please observe and record any patient safety hazards you see but do not communicate with each other or physically interact with the simulated environment. Your responses will be anonymous. You will have ten minutes inside the room.”
